# The Burden of Cerebral Venous Thrombosis in a Romanian Population across a 5-Year Period

**DOI:** 10.3390/life12111825

**Published:** 2022-11-09

**Authors:** Adina Stan, Silvina Ilut, Hanna Maria Dragos, Claudia Bota, Patricia Nicoleta Hanghicel, Alexander Cristian, Irina Vlad, Diana Mocanu, Stefan Strilciuc, Paul Stefan Panaitescu, Horatiu Stan, Dafin F. Muresanu

**Affiliations:** 1 Department of Neurosciences, Iuliu Hatieganu University of Medicine and Pharmacy, No. 8 Victor Babes Street, 400012 Cluj-Napoca, Romania; 2 RoNeuro Institute for Neurological Research and Diagnostic, No. 37 Mircea Eliade Street, 400364 Cluj-Napoca, Romania; 3 Neurology Department, Emergency County Hospital Cluj-Napoca, 400347 Cluj-Napoca, Romania; 4 Department of Microbiology, Iuliu Hatieganu University of Medicine and Pharmacy, No. 8 Victor Babes Street, 400012 Cluj-Napoca, Romania

**Keywords:** cerebral venous thrombosis, incidence, mortality, length of stay

## Abstract

Health policies in transitioning health systems are rarely informed by the social burden and the incidence shifts in disease epidemiology. Cerebral venous thrombosis (CVT) is a type of stroke more often affecting younger adults and women, with higher incidences being reported in recent studies. A retrospective, hospital-based population study was conducted at Cluj-Napoca Emergency County Hospital across a 5-year period between 2017 and 2021. The overall incidence and the rates in distinctive gender and age groups were assessed. Length of hospital stay (LHS), modified Rankin score (mRS) and mortality at discharge and at 3 months were calculated. Fifty-three patients were included. The median age was 45 years, and 64.2% were women. In our population of 3,043,998 person-years, 53 CVT cases resulted in an incidence of 1.74 per 100,000 (95% CI 1.30–2.27). CVT incidence was higher in women (2.13 per 100,000, 95% CI 1.47–2.07). There was a statistically significant difference in LHS between patients with different intracranial complications (Kruskal–Wallis, *p* = 0.008). The discharge mRS correlated with increasing age (r_s_ = 0.334, *p* = 0.015), transient risk factors (Fisher’s exact test, *p* = 0.023) and intracranial complications (Fisher’s exact test, *p* = 0.022). In addition, the mRS at 3 months was statistically associated with increasing age (r_s_ = 0.372, *p* = 0.006) and transient risk factors (Fisher’s exact test, *p* = 0.012). In-hospital mortality was 5.7%, and mortality at follow up was 7.5%, with higher rates in women (5.9% and 8.8%, respectively). Our findings may provide insight regarding the epidemiological features of certain patient groups more prone to developing CVT and its complications, informing local and central stakeholders’ efforts to improve standards of care.

## 1. Introduction

Cerebral venous thrombosis (CVT) is an uncommon type of stroke that affects younger adults and women more often than ischemic and hemorrhagic stroke [1,2,3]. CVT incidence was previously estimated at around 0.2 to 0.5 per 100,000 per year [4,5], but higher incidences of 1.57 or 1.32 per 100,000 have been reported in recent studies [3,6,7]. Whether the accessibility to neuroimaging diagnosis and methodological differences between studies may explain these findings or a true increase in incidence was identified in the last decade is not clear. No studies on CVT incidence in Romanian populations were found.

First estimates of mortality in CVT patients are derived from autopsy studies performed several decades ago [8]. In the past, CVT was associated with poor prognosis and high mortality rate [8]. In recent studies [6,7,9,10,11,12], the prognosis became more favorable; a mortality of 4% to 5% in the acute phase and a declining trend in overall mortality among patients with CVT have been described [3,13]. The majority of data on CVT patients was derived from reference cohorts such as the multicenter prospective and retrospective VENOST study [14] and the International Study on Cerebral Vein and Dural Sinus Thrombosis (ISCVT) [1], where a discharge mortality of 4.3% and a mortality of 8.3% at follow up were reported [1]. Inconsistent with previous studies [6,7,9,10,15,16], no fatal events were reported among a Romanian cohort of 43 patients in 2014 [11].

Our study was designed to address these inconsistencies as well as the lack of local epidemiological data. The primary aim was to investigate the incidence and the social burden of CVT on a Romanian-hospital-based population, by assessing the length of hospital stay (LHS), modified Rankin scale (mRS) and mortality at discharge and at three months. The secondary aim was to perform a literature review, comparing our findings with those reported by other hospital-based population studies on incidence and mortality in CVT patients.

## 2. Materials and Methods

A retrospective hospital-population-based study was conducted at Cluj-Napoca Emergency County Hospital (CNECH), the second largest tertiary stroke center in Romania. Patients were identified through electronic charts based on the relevant International Classification of Diseases, Tenth Revision (ICD-10) codes for CVT cases during a 5-year period between 2017 and 2021. Only inhabitants living in the hospital’s catchment area (Cluj County) were included. The hospital had a catchment area of around 608,800 habitants/year between 2017 and 2021. All CVT cases in this area are in contact with our hospital at the initial admission as part of acute management and follow up. Thus, it is possible to estimate population-based rates for CVT. Population figures for the incidence rates were obtained from the Romanian National Institute of Statistics [17].

The hospital’s electronic database was searched to identify patients over 18 years old with a new CVT diagnosis between 1 January 2017 and 31 December 2021. The following ICD-10 codes were searched for: I63.6, I67.6, O22.5 and O87.3. All clinical and neuroradiological assessments for the identified CVT cases were reassessed and confirmed by a senior neurologist. Patients with repeated presentations due to chronic CVT during the study period were counted as one case in the analysis. Demographic, clinical, radiological and potential risk factors were investigated for all patients.

To calculate the overall incidence, the observed CVT cases admitted to CNECH were used as the numerator and the population of Cluj County aged ≥ 18 years as the denominator. The adult population of Cluj County was 604,635 in 2017, 606,485 in 2018, 608,739 in 2019, 611,508 in 2020 and 612,631 in 2021 [17], resulting in 3,043,998 person-years across the 5-year period. In addition, the CVT incidences depending on gender and different age groups were also assessed. Incidence rates were expressed per 100,000 person-years, and 95% confidence intervals (95% CI) were displayed.

To assess the burden of CVT at the regional level, LHS, mRS and mortality at discharge and at three months were calculated. The influence of the main predictive factors for outcome in CVT [1] was also investigated.

Since the sample size was narrow, determining the distribution of continuous variables was important for choosing an appropriate statistical method. As the LHS variable showed a positively skewed distribution (Shapiro–Wilk test, *p* = 0.001), the median and the interquartile range were used to describe these data. Logarithmic transformations [18] for LHS were performed, but the data became more skewed, departing from normal distribution (Shapiro–Wilk test, *p* < 0.001); therefore, non-parametric tests were selected. Spearman correlation was used to investigate the association between age and LHS, mRS at discharge or mRS at three months. Categorical data were presented as counts and percentages. The differences between groups of categorical data were assessed using chi-square or Fisher’s exact test, and the effect size was described using Crammer’s V values. The significance level alpha was 0.05. Statistical analyses were performed using IBM SPSS Statistics, version 26.0. The study protocol was approved by the Independent Ethics Committee of CNECH.

## 3. Results

### 3.1. Study Population

Among the 53 patients included, the median age was 45 years (range: 18–89), and 64.2% were women. Table 1 shows the demographics, risk factors and clinical and radiological findings among all included patients. In all, 60.4% of patients presented with acute onset. The most prevalent symptom was headache, followed by motor weakness, seizures and nausea/vomiting. Most patients developed CVT involving two or more sinuses/veins. Among those with only one sinus involved, the most frequent sites were the transverse sinus and the superior sagittal sinus. CVT risk factors were found in 75.47% of the patients, and most patients had at least one identified risk factor. The most frequent transient risk factors in women were pregnancy or puerperium and oral contraceptive use, whereas head trauma and local infections were the only transient risk factors encountered in men. Thrombophilia was by far the most common persistent risk factor in all patients. No transient or persistent risk factors were found in thirteen patients. Furthermore, 18.8% of patients presented at least two intracranial lesions on CT or MRI. Venous infarction was the most common radiological finding among all patients. Parenchymal hemorrhage was more frequent in men and subarachnoid hemorrhage in women.

### 3.2. The Incidence of CVT

**Figure 1:**
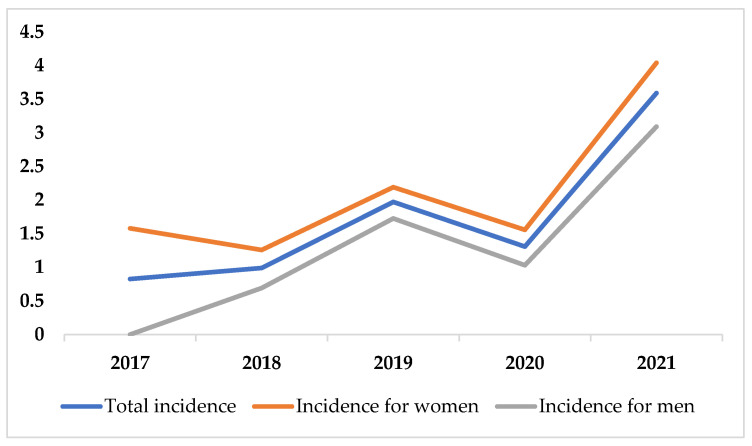
Incidence of CVT across 5-year period.

In our population of 3,043,998 person-years, 53 identified cases resulted in an incidence of 1.74 per 100,000 (95% CI 1.30–2.27). The incidence of CVT across a 5-year period and incidence figures for different gender and age groups are depicted in Figure 1 and in Table 2, respectively. CVT incidence was higher in women compared to men. The highest incidence of CVT was found in 2021 (3.59 per 100,000, 95% CI 2.25–5.43). Across the 5-year period, CVT incidence seems to follow a general ascending trend; although, in 2020, a decline in incidence was registered. The CVT incidence in patients between 18 and 49 years old increased 4.5-fold in 2021 compared to 2017. The highest overall incidence among all gender and age groups was found for women in the 18–49 age group (2.43 per 100,000, 95% CI 1.43–3.62). Only in the 50–69 age group, the incidence rates for men and women were similar.

### 3.3. The Burden of CVT

The median LHS was 10 days (interquartile range 7). There was a statistically significant difference in LHS between patients with different intracranial lesions (Kruskal–Wallis, χ^2^(4) = 13.906, *p* = 0.008), with a mean rank of LHS of 17.44 for venous infarct patients, 24 for subarachnoid hemorrhage patients, 41.83 for intracranial hemorrhage patients and 39.6 in patients who had developed multiple complications. No associations between LHS and age (r_s_ = 0.077, *p* > 0.05), gender (Mann–Whitney, *p* > 0.05), CVT location, risk factors or mRS were found (Kruskal–Wallis, *p* > 0.05).

The median of mRS at discharge was 2 (interquartile range 2). A positive moderate relationship between discharge mRS and age was seen (r_s_ = 0.334, *p* = 0.015), indicating that 11.15% of the variation of discharge mRS was explained by age (r_s_^2^ = 0.1115). Moreover, there was a significant difference in discharge mRS between patients with different transient risk factors (Fisher’s exact test, *p* = 0.023, Cramer’s V of 0.473, medium effect size). Women in pregnancy/puerperium or using oral contraceptive had an mRS between 0 and 3. Patients with traumatic brain injury presented a higher mRS of 2–4, and 50% of patients with infections died during hospitalization. A significant association was found between discharge mRS and intracranial lesions (Fisher’s exact test, *p* = 0.022, Cramer’s V of 0.434, medium effect size). Most patients with venous infarction had an mRS of 0–2, while those who developed multiple lesions had an mRS > 2 at discharge. Meanwhile, 66.7% of patients with intraparenchymal hemorrhage had a discharge mRS of 4. No statistical associations between mRS and gender, CVT location or persistent risk factors were found (chi-square, *p* > 0.05).

**Figure 2:**
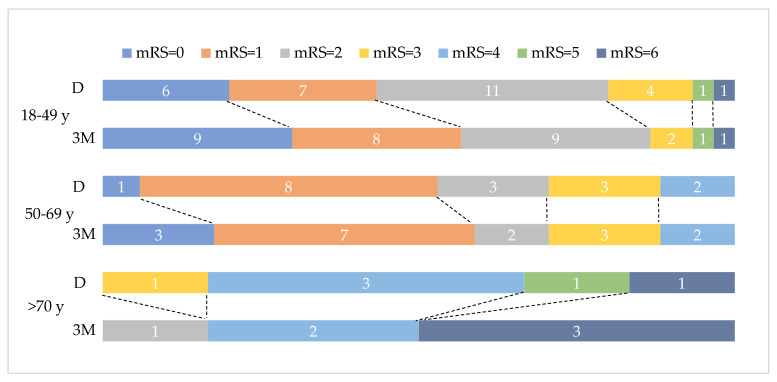
Discharge (D) and three-month (3M) mRS distribution on age groups. Data labels represent number of patients per mRS group.

The median of mRS at follow up was 1 (interquartile range 2). A positive moderate relationship between 3-month mRS and age was seen (r_s_ = 0.372, *p* = 0.006), indicating that 13.83% of the variation of 3-month mRS was explained by age (r_s_^2^ = 0.1383). A significant association between mRS and transient risk factors was found (Fisher’s exact test, *p* = 0.012, Cramer’s V of 0.461, medium effect size). Most patients without transient risk factors had a 3-month mRS between 0 and 2. No statistical associations between 3-month mRS and gender, CVT location, intracranial lesions or persistent risk factors were found (chi-square, *p* > 0.05). Figure 2 depicts the distribution of mRS at discharge and at three months among different age groups.

In-hospital mortality was 5.7% for the total sample, with higher rates in women compared to males. The >70 age group showed the highest discharge mortality (16.7%). Mortality at follow up was 7.5%. Three-month mortality was higher for women. A significant association between mortality at follow up and age groups was found (Fisher’s exact test, *p* = 0.003, Cramer’s V of 0.577, strong effect size). Overall, 50% of patients over 70 years old died at 3 months after CVT compared to 3.3% of patients between 18 and 49 years old. No patients from the 50–69 years age group died in the first three months.

Table 3 displays our results regarding the incidence and mortality of CVT in relation to previous hospital-based population studies in different countries. Based on the country of origin, studies were classified as coming from high-, upper- or lower-middle-income countries using the definition of the World Bank [19]. Among high-income countries, an increasing trend in incidence and a decline in mortality at discharge and follow up were identified across the last 40 years. A tendency for increased age at CVT diagnosis could be observed. Fewer and heterogenous data from upper- and lower-middle-income countries were found. The median age and proportion of women from our study were similar to those reported by the VENOST study [14], but the overall incidence found in our cohort was comparable to recent incidences observed in studies in high-income countries.

## 4. Discussion

### 4.1. The Incidence of CVT

The overall incidence of CVT found in this study corroborates those of other recent studies from Norway [9], Italy [12], Australia [6] and two Dutch [7] provinces, reporting higher annual CVT incidence than previous studies [15,16,21].

CVT incidence varies in distinct parts of the world due to distinctive socioeconomic and demographic features or risk factors [9]. A cohort derived from four prospective international studies [22] showed that the incidence of venous thromboembolism was higher in high-income countries compared to upper-middle-income countries and lower-middle-/low-income countries. The diagnosis of thrombosis events seems to be affected by reduced access to hospital and diagnostic facilities in low-income countries [22]. Regarding Romania, the increasing accessibility to CT and MRI venography may explain the higher incidence comparable to that reported by high-income countries.

In our study, the incidence for women was higher compared with other studies (2.13 per 100,000 person-years, 95% CI 1.47–2.97) [6,9,10,12,21]. It was shown that the CVT risk in women using oral contraceptives is 7.59 times higher compared to that in women not taking oral contraception [23]. Combined oral contraceptives (COCs) represented 8.8% of contraceptive use prevalence, reaching 15.4% or more in high-income countries [24]. A cross-sectional survey [25] on COC use conducted in 2014 on women in community pharmacies in Cluj-Napoca revealed that 38.9% of women had used COCs for more than two years, out of which more than half bought the pills without prescription from a pharmacy at least once [25], raising concerns about harmful use.

A recent meta-analysis [23] on pregnancy-related strokes reported an incidence of 9.1 cases of CVT per 100,000 pregnancies, with CVT accounting for from 9% up to 48% of total pregnancy-related strokes. In our study, 11.8% of patients presented pregnancy-related CVT, half of them occurring in the first two postpartum weeks. Interestingly, the other half of patients developed CVT in the first trimester, which is exceedingly rare. Incongruent with other studies [23,26], the patients had associated complications such as bilateral thalamic infarction and parenchymal hemorrhages.

In the 50–69 age group, the incidence rates for men and women were equal, similar to other studies reporting no predilection toward women in elderly CVT patients [2,27,28,29]. This finding could be explained by a decreasing trend in CVT incidence in women aged 50–69, attributed to the absence of gender-specific risk factors, especially COCs and pregnancy/puerperium state.

The lowest incidence of CVT was found in men over 70 years old probably due to a poor life expectancy in men compared to women (70 years old versus 79 years old) and an overrepresentation of women among all age groups (171,104 men compared to 264,374 women reported across the 5-year analyzed period) [17]. An important clinical finding in multiple studies [27,29] on CVT in older patients is the absence of headache in patients over 55 years old, but in our study, no significant difference between different age groups and the presence of headache or other CVT symptoms was found (chi-square, *p* > 0.05).

### 4.2. The Burden of CVT

In our study, the median LHS was 10 days, which was similar to the median LHS found in a French study [10], but much lower compared to that in the ISCVT [1] study probably due to different study periods (1998–2002 in ISCVT versus 2017–2021 in our study). A decreased LHS was reported by recent American studies (median LHS = 4) [30,31], with longer LHS being associated with age and male gender. In our study, no association between LHS and age, gender, CVT location, risk factors or outcome was found.

Discharge mRS increased with age, which is similar to the case in previous studies [1,14]. Half of the patients who had any infections (nose, throat, ear or extracranial) as the cause of their CVT died, and patients who developed CVT after previous cranial trauma had an mRS of 2 to 4 at discharge (Fisher’s exact test, *p* = 0.023). These findings are similar to those of the ISCVT cohort [1] that reported a 3.34-fold increased risk of death or dependency in patients with infections and CVT. Patients with two or more intracranial complications had an mRS > 2 at discharge. Furthermore, 66.7% of patients with parenchymal hemorrhage presented an mRS of 4; these results are also similar to those reported by ISCVT investigators [1].

Risk factors associated with poor prognosis at 3 months were similar to those found in acute phase: age, infections and cranial trauma. Neither parenchymal hemorrhage nor persistent risk factors such as cancer influenced the outcome at 3 months (chi-square, *p* > 0.05), which is different from ISCVT [1] and VENOST [14] studies, which found that any malignancy is associated with a 2.9 increased risk of death or dependency [1].

The in-hospital mortality rate was 5.7%, similar to that in previous hospital-based studies [6,7,9,10,15,16,20,21]. In the ISCVT [1], a discharge mortality rate of 4.3% and mortality of 8% at follow up were reported. Among high-income countries [6,7,9,10,15,16] (Table 3), a trend in declining mortality in adult patients diagnosed with CVT was observed at least in the last decade. A significant inverse correlation between mortality and year of patient recruitment (r = −0.72, *p* < 0.001) was reported [13]. The sensitivity analysis of the studies from high-income countries reported a similar inverse correlation (r = −0.70, *p* < 0.001) [13]. Interestingly, after the exclusion of studies published before 1990, the inverse correlation persisted (r = −0.51, *p* < 0.001), but when all studies published before 2000 were excluded, the correlation disappeared (r = −0.06, *p* = 0.67) [13].

Part of the decline in mortality is due to the general improvement of hospital care and increasing availability of neuroimaging techniques and recent therapeutic strategies. Before the implementation of cerebral angiography, CVT was diagnosed with certainty only at surgery or autopsy, with the selection bias of patients in severe clinical condition [13,32] being common in the first mortality studies. The severity of CVT cases has also decreased over time, with fewer cases of coma or severe neurological deficits being identified [7,13,21]. Moreover, the introduction of anticoagulation [33] and decompressive hemicraniectomy [34] improved the survival of patients. A shift in risk factors associated with CVT may also explain the decline in mortality and increase in incidence. Across the years, traumatic and septic CVT have decreased, while the number of women using oral contraceptives increased [13]. It is known that trauma- or sepsis-related CVT has a worse prognosis [35] compared to a favorable outcome in patients using oral contraceptives [2] as was found in our cohort.

It is important to observe shifts in the pattern of incidence and mortality in patients with CVT, also considering the pre-pandemic and pandemic periods. CVT is a rare but severe complication after SARS-CoV-2 infection [36,37,38], and CVT has been reported following immunization [39], especially with adenovirus-vector-based [40] and ChAdOx1-S vaccines [41]. Our sample included a patient with a moderate form of COVID-19 infection–associated CVT and a patient who developed CVT after 10 days from adenovirus-vector-based SARS-CoV-2 vaccination. CVT occurring after adenoviral-vector-based COVID-19 vaccination is usually associated with vaccine-induced immune thrombotic thrombocytopenia and the presence of antibodies against platelet factor 4 (PF4) [42,43], but the platelet counts of our patient were normal across multiple blood tests, and PF4 antibodies were not evaluated.

### 4.3. Limitations

The demographics, clinical features and associated risk factors suggest that our study included a sample of patients that is representative for CVT [1]. The sample size may be regarded as narrow with 53 cases, but the study extended across five years and screened all acute cerebrovascular presentations at the second-largest stroke center in Romania. Another limitation is the retrospective design of the study, but because an extensive assessment of the anonymized electronic medical records was performed, it is unlikely that this design might have introduced bias.

## 5. Conclusions

This study investigated the burden of CVT in a Romanian-hospital-based population and compared regional findings with cross-sectional studies from different countries. These results have clinical practice implications concerning additional investigations and the management and prognosis of CVT. The diagnosis of one subsequent disease or risk factor in CVT patients should not limit the search for additional comorbidities or risk factors. Furthermore, as infections, cranial trauma and intracranial hemorrhage are potential predictors of poor outcome, patients showing these features should be thoroughly investigated and monitored. CVT shows an increasing incidence particularly in the 18–49 age group in the Romanian population across the last five years. In the presence of suggestive CVT symptoms in this age group, a CT angiography at the emergency department should be considered to exclude CVT. Further multicenter prospective studies on Romanian populations should be conducted to assess the incidence and the burden of CVT.

## Figures and Tables

**Table 1:** Demographic, clinical, radiological and risk factor data presented as total and percentage.

Patient Characteristics	Total, N = 53	Female, N = 34, 64.2%	Male, N = 19, 35.8%
Median age (interquartile range), years	45 (29)	45 (22)	44 (40)
Type of onset, N (%)			
Acute	32 (60.4%)	20 (58.8%)	12 (63.2%)
Subacute	16 (30.2%)	10 (29.4%)	6 (31.6%)
Chronic	5 (9.4%)	4 (11.8%)	1 (5.2%)
Clinical presentation, N (%)			
Headache	31 (58.5%)	22 (64.7%)	9 (47.4%)
Nausea/vomiting	10 (18.9%)	6 (17.6%)	4 (21.1%)
Motor weakness	19 (35.8%)	14 (41.2%)	5 (26.3%)
Seizures	14 (26.4%)	6 (17.6%)	8 (42.1%)
Coma	9 (17%)	6 (17.6%)	3 (15.8%)
Speech disturbances	6 (11.3%)	6 (17.6%)	0
Cranial nerve palsies	5 (9.43%)	2 (5.8%)	3 (15.8%)
Cerebellar signs	4 (7.5%)	4 (11.8%)	0
Sinus/vein involved, N (%)			
Transverse sinus	8 (15.1%)	6 (17.6%)	2 (10.5%)
Superior sagittal sinus	5 (9.4%)	2 (5.9%)	3 (15.8%)
Cavernous sinus	3 (5.7%)	0	3 (15.8%)
Cortical veins	3 (5.7%)	3 (8.8%)	0
Two or more sinuses/veins involved	34 (64.1%)	23 (67.7%)	11 (57.9%)
Transient risk factors, N (%)			
Pregnancy and puerperium	4 (7.5%)	4 (11.8%)	0
Oral contraceptives	4 (7.5%)	4 (11.8%)	0
Head trauma	6 (11.3%)	3 (8.8%)	3 (15.8%)
Local infections	4 (7.5%)	2 (5.9%)	2 (10.5%)
SARS-CoV-2 infection	1 (1.88%)	1 (2.94%)	0
Adenoviral-vector-based SARS-CoV-2 vaccination	1 (1.88%)	0	1 (5.2%)
Two or more transient risk factors	2 (3.8%)	2 (5.9%)	0
Persistent risk factors, N (%)			
Prior thromboembolism	3 (5.7%)	2 (5.9%)	1 (5.3%)
Thrombophilia	13 (24.5%)	7 (20.6%)	6 (31.6%)
Neoplasia	4 (7.5%)	4 (11.7%)	0
Two or more persistent risk factors	7 (13.2%)	5 (14.7%)	2 (10.5%)
Without transient or persistent risk factors, N (%)	13 (24.52%)	7 (20.56%)	6 (31.6%)
Complications, N (%)			
Venous infarct	9 (17%)	7 (20.6%)	2 (10.5%)
Subarachnoid hemorrhage	3 (5.7%)	2 (5.9%)	1 (5.3%)
Parenchymal hemorrhage	3 (5.7%)	0	3 (15.7%)
Two or more complications	10 (18.8%)	9 (26.4%)	1 (5.3%)
Without complications	28 (52.8%)	16 (47.1%)	12 (63.2%)

**Table 2:** Incidence of CVT across the 5-year period regarding gender and age groups (95% CI).

	2017	2018	2019	2020	2021	Overall Incidence
Incidence/year	0.82 (0.26–1.92)	0.98 (0.36–2.15)	1.97 (1.09–3.44)	1.30 (0.56–2.57)	3.59 (2.25–5.43)	1.74 (1.30–2.27)
Women	1.57 (0.51–3.68)	1.25 (0.34–3.22)	2.19 (0.88–4.51)	1.55 (0.50–3.63)	4.04 (2.12–6.91)	2.13 (1.47–2.97)
Men	0	0.69 (0.08–2.50)	1.72 (0.56–4.03)	1.03 (0.21–3.01)	3.09 (1.41–5.87)	1.31 (0.79–2.04)
Incidence/18–49 y	0.86 (0.17–2.52)	1.75 (0.64–3.81)	1.77 (0.65–3.85)	0.59 (0.07–2.15)	3.89 (2.07–6.66)	1.76 (1.19–2.53)
Women	1.72 (0.35–5.04)	2.32 (0.63–5.95)	1.76 (0.36–5.15)	0.59 (0.01–3.29)	5.36 (2.45–10.1)	2.43 (1.43–3.62)
Men	0	1.17 (0.01–4.23)	1.77 (0.03–5.19)	0.59 (0.01–3.33)	2.41 (0.06–6.17)	1.18 (0.05–2.17)
Incidence/50–69 y	0.57 (0.01–3.20)	0	2.18 (0.05–5.58)	2.67 (0.08–6.25)	3.71 (1.49–7.64)	1.86 (1.08–2.08)
Women	1.09 (0.02–6.07)	0	2.07 (0.02–7.47)	4.07 (1.11–10.4)	2.01 (0.02–7.29)	1.87 (0.08–3.56)
Men	0	0	2.30 (0.02–8.32)	1.13 (0.02–6.29)	5.58 (1.82–13.0)	1.85 (0.80–3.65)
Incidence/>70 y	1.18 (0.03–6.61)	0	2.31 (0.28–8.34)	1.12 (0.28–6.26)	2.20 (0.26–7.98)	1.37 (0.50–2.99)
Women	1.95 (0.49–10.8)	0	3.80 (0.46–13.7)	0	3.64 (0.44–13.1)	1.89 (0.61–4.44)
Men	0	0	0	2.85 (0.72–15.8)	0	0.58 (0.14–3.25)

**Table 3:** Different studies reporting the incidence and the mortality of CVT on local populations compared to ISCVT.

	ISCVT [1]	High-Income Countries							Upper-Middle-Income Countries			Lower-Middle-Income Countries
Country	21 Countries	Portugal [15]	Hong Kong [16]	Australia [6]	Netherlands [7]	France [10]	Norway [9]	Italy [12]	Mexico [20]	VENOST [14], Turkey	Romania (Our Study)	Iran [21]
Time interval	1998–2002	1980–1998	1995–1998	2005–2011	2008–2010	2011–2016	2011–2017	2012–2019	1999–2008	2000–2015	2017–2021	2001–2004
Sample size	624	142	13	105	94	194	62	32	24	1144	53	122
Incidence per 100,000	NR	0.22	0.34	1.57	1.32	NR	1.75	1.6	NR	NR	1.74	1.23
Median age	37 (16–86)	35	30	49	41	40	46	41	30	40	45	26
Women, %	74.5%	72%	77%	52%	72%	68.4%	53%	75%	83%	67.9%	64.2%	79%
LHS, median	17	NR	NR	NR	NR	10	NR	20	22	NR	10	NR
Discharge mortality	4.3%	6%	8%	9%	1%	2.9%	3%	0%	8%	NR	5.7%	NR
Mortality at follow up	8.3%	NR	NR	12%	3%	3.4%	3%	0%	NR	NR	7.5%	NR

ISCVT = International Study on Cerebral Vein and Dural Sinus Thrombosis, NR = not reported.

## Data Availability

Data available on request due to privacy or ethical issues. The data presented in this study are available on request from the corresponding author.
